# A Priori Prediction of Neoadjuvant Chemotherapy Response in Breast Cancer Using Deep Features from Pre-Treatment MRI and CT

**DOI:** 10.3390/cancers17203394

**Published:** 2025-10-21

**Authors:** Deok Hyun Jang, Laurentius O. Osapoetra, Lakshmanan Sannachi, Belinda Curpen, Ana Pejović-Milić, Gregory J. Czarnota

**Affiliations:** 1 Physical Sciences, Sunnybrook Research Institute, Toronto, ON M4N 3M5, Canada; 2 Department of Radiation Oncology, Sunnybrook Health Sciences Centre, Toronto, ON M4N 3M5, Canada; 3 Department of Physics, Toronto Metropolitan University, Toronto, ON M5B 2K3, Canada; 4 Department of Medical Biophysics, Faculty of Medicine, University of Toronto, Toronto, ON M5T 1P5, Canada; 5 Department of Medical Imaging, Sunnybrook Health Sciences Centre, Toronto, ON M4N 3M5, Canada; 6 Department of Radiation Oncology, Faculty of Medicine, University of Toronto, Toronto, ON M5T 1P5, Canada

**Keywords:** radiomics, MRI, CT, breast cancer, neoadjuvant chemotherapy, response prediction, machine learning, deep learning

## Abstract

**Background:** Response to neoadjuvant chemotherapy (NAC) is a key prognostic indicator in breast cancer, yet current assessment relies on postoperative pathology. This study investigated the use of deep features derived from pre-treatment MRI and CT scans, in conjunction with clinical variables, to predict treatment response a priori. **Methods:** Two response endpoints were analyzed: pathologic complete response (pCR) versus non-pCR, and responders versus non-responders, with response defined as a reduction in tumor size of at least 30%. Intratumoral and peritumoral segmentations were generated on contrast-enhanced T1-weighted (CE-T1) and T2-weighted MRI, as well as contrast-enhanced CT images of tumors. Deep features were extracted from these regions using ResNet10, ResNet18, ResNet34, and ResNet50 architectures pre-trained with MedicalNet. Handcrafted radiomic features were also extracted for comparison. Feature selection was conducted with minimum redundancy maximum relevance (mRMR) followed by recursive feature elimination (RFE), and classification was performed using XGBoost across ten independent data partitions. **Results:** A total of 177 patients were analyzed in this study. ResNet34-derived features achieved the highest overall classification performance under both criteria, outperforming handcrafted features and deep features from other ResNet architectures. For distinguishing pCR from non-pCR, ResNet34 achieved a balanced accuracy of 81.6%, whereas handcrafted radiomics achieved 77.9%. For distinguishing responders from non-responders, ResNet34 achieved a balanced accuracy of 73.5%, compared with 70.2% for handcrafted radiomics. **Conclusions:** Deep features extracted from routinely acquired MRI and CT, when combined with clinical information, improve the prediction of NAC response in breast cancer. This multimodal framework demonstrates the value of deep learning-based approaches as a complement to handcrafted radiomics and provides a basis for more individualized treatment strategies.

## Simple Summary

Early identification of breast cancer patients who are unlikely to respond to neoadjuvant chemotherapy (NAC) is critical for potentially guiding alternative therapeutic strategies. In this study, routinely acquired pre-treatment MRI and CT scans were analyzed using deep learning-based features in combination with clinical information. Deep features were extracted from intratumoral and peritumoral regions using ResNet architectures pre-trained on large-scale medical imaging datasets. Among the models tested, ResNet34 demonstrated the best performance, exceeding both handcrafted radiomic models and other ResNet backbones. These findings suggest that deep features extracted from standard-of-care imaging can complement established clinical predictors and may facilitate more personalized treatment planning in breast cancer.

## 1. Introduction

Breast cancer is a highly heterogeneous disease characterized by significant genetic, phenotypic, and microenvironmental diversity at both the intertumoral and intratumoral levels [1]. This heterogeneity leads to variability in treatment efficacy and patient prognosis. Consequently, breast cancer management employs a multimodal approach that integrates locoregional treatments, such as surgery and radiation therapy, with systemic therapies including chemotherapy, targeted therapy, immunotherapy, and endocrine therapy [2,3]. In the treatment of locally advanced breast cancer (LABC) and high-risk early breast cancer (EBC), chemotherapy is typically administered in the neoadjuvant setting before surgery [4,5]. The aim of neoadjuvant chemotherapy (NAC) is to downstage the tumor, improve operability, and eradicate micrometastases.

Response to NAC refers to tumor reduction following therapy and serves as an indicator of therapeutic efficacy. In particular, pathological complete response (pCR), defined as the complete clearance of invasive carcinoma in the breast and lymph nodes, is associated with improved survival outcomes [6,7]. This effect is more pronounced in human epidermal growth factor receptor 2 (HER2)-enriched and triple-negative breast cancers [8]. Due to its clinical significance, pCR is recognized by the American and European regulatory agencies as a surrogate endpoint for long-term outcomes in randomized clinical trials [9,10]. Nonetheless, the likelihood of achieving pCR is relatively low, as a meta-analysis of 52 studies involving 27,895 patients reported a pCR rate of 21.1% [8]. The majority of patients receiving NAC achieve a partial response, defined as tumor shrinkage with residual invasive disease, which corresponds to intermediate survival outcomes between pCR and stable or progressive disease [11,12].

Given the prognostic significance of NAC response, the early identification of less responsive patients could enable timely treatment modification to improve the likelihood of response and survival outcomes [13]. However, the gold standard for assessing response remains histopathological examination of the surgical specimen, which is only available after the completion of NAC and subsequent surgery. Imaging modalities such as magnetic resonance imaging (MRI) and computed tomography (CT) acquired during NAC may provide insights into tumor response to NAC at an early stage. However, such interim imaging is not widely established as a standard protocol in breast cancer management [2]. As a result, pre-treatment imaging has been increasingly investigated for response prediction using radiomic analysis. Radiomics involves the extraction of high-dimensional quantitative features from medical images, capturing patho-physiological information not discernible by visual assessment [14,15]. In breast cancer, radiomics can effectively quantify intratumoral heterogeneity. The utility of radiomics for predicting breast cancer response has been investigated using various imaging modalities, such as quantitative ultrasound [16,17], CT [18,19,20,21], and MRI [22,23,24].

Conventionally, radiomic analysis has relied on predefined mathematical descriptors, often referred to as handcrafted features, which are subsequently used in machine learning classification. Deep learning offers an alternative that integrates the learning of layered image representations directly from imaging data with classification in a single framework, while eliminating the need for explicit radiomic feature extraction and feature selection [25,26,27]. However, the effectiveness of end-to-end deep learning models is often constrained by the limited size of annotated medical imaging datasets, which can lead to overfitting and poor generalizability [28]. To address this limitation, deep feature extraction has been explored as a hybrid strategy that utilizes intermediate activations from deep networks as input features for traditional machine learning classifiers [27,29]. Transfer learning, which adapts models pretrained on large datasets to new tasks with limited data, can further improve this approach by providing more robust feature representations. In particular, pretrained weights derived from large-scale medical imaging datasets such as MedicalNet [30] have the potential to enhance the stability and generalizability of the extracted deep features. These approaches leverage the ability of convolutional neural network (CNN) to capture complex image patterns that may correspond to underlying biological heterogeneity not represented by handcrafted radiomic features, while avoiding the substantial data requirements and heavy computational burden of fully trainable deep learning models. Therefore, deep feature extraction offers a balanced approach that combines the strengths of both radiomics and deep learning and may provide improved predictive performance in breast cancer response modeling.

This study aimed to predict breast cancer response to NAC using a deep feature extraction framework with transfer learning. Two different binary classifications of response were assessed: pCR versus non-pCR, and clinical response versus non-response. In this context, clinical response was defined to include both complete and partial responders. The analysis utilized routinely acquired pre-treatment scans obtained as part of standard diagnostic work-up, including contrast-enhanced T1-weighted (CE-T1) and T2-weighted (T2) MRI sequences and contrast-enhanced CT images. Intratumoral and peritumoral segmentations were analyzed to capture both tumor-intrinsic and surrounding microenvironmental characteristics relevant to therapy response. Deep feature extraction was implemented using CNNs with transfer learning from pre-trained medical imaging models, and the resulting features were subsequently applied to machine learning classifiers. This approach provides a balance between the interpretability of handcrafted radiomics and the representational capacity of deep learning, while reducing dependency on large, annotated cohorts. Ultimately, incorporating deep features from routine imaging may improve prediction of NAC response and, in turn, support future individualized treatment strategies in breast cancer management.

## 2. Materials and Methods

### 2.1. Patient Selection

This study was conducted at Sunnybrook Health Sciences Centre, Toronto, Canada, with institutional research ethics board approval. Patients with biopsy-confirmed breast cancer diagnosed between 2013 and 2019 were reviewed. Eligible cases for imaging data analysis included individuals with LABC or high-risk early-stage disease who underwent standard anthracycline- and taxane-based NAC followed by surgery. Patients were treated with one of two standard NAC regimens. In the first regimen, three cycles of fluorouracil, epirubicin, and cyclophosphamide were administered, followed by three cycles of docetaxel given at three-week intervals. In the alternative dose-dense protocol, four cycles of doxorubicin and cyclophosphamide were administered, followed by four cycles of paclitaxel every two weeks. Patients with HER2-enriched disease were additionally treated with trastuzumab as targeted therapy. Availability of biopsy results, pre-treatment MRI and CT images, and surgical pathology after NAC was required for inclusion in the study. Patients were excluded if images were of insufficient quality, contained artifacts, or had incomplete coverage of the primary tumor. Cases with missing biopsy or surgical pathology reports or with breast implants were also excluded. All patient information was anonymized prior to analysis.

### 2.2. Clinical Variables

Clinical characteristics were included in the modeling to account for established prognostic and predictive factors in breast cancer. Estrogen receptor (ER), progesterone receptor (PR), and HER2 status were determined from biopsy by immunohistochemistry (IHC) and incorporated as binary variables. Additional variables consisted of patient age at diagnosis, histological grade (categorized as G1, G2, or G3), primary tumor size in millimeters measured on pre-treatment MRI, and clinical nodal status (categorized as N0 to N3 according to AJCC staging). Altogether, seven clinical features were integrated with imaging-derived features for predictive analysis. Age and tumor size were continuous variables, and the remaining features were categorical.

### 2.3. Response Assessment

Two definitions of response were evaluated as treatment outcomes following NAC. In criterion 1, the response was defined as pCR based on histopathological examination of the surgical specimen, regardless of the presence of ductal carcinoma in situ (DCIS). Therefore, criterion 1 distinguished between pCR and non-pCR, with the latter encompassing both partial response and non-response. In criterion 2, the response was defined as a reduction in tumor size of 30% or greater, based on a modified grading system [31] adapted from the Response Evaluation Criteria in Solid Tumors (RECIST) guidelines [32]. The initial tumor size was measured as the longest diameter on pre-treatment MRI, and post-treatment tumor size was obtained from surgical pathology. This indirect comparison was necessary because post-treatment MRI was not routinely acquired at the institution. In this definition, the response included both complete and partial response, whereas the non-response included stable or progressive disease. Additionally, residual tumor cellularity of less than 1% was also interpreted as an indicator of treatment response.

### 2.4. Image Acquisition and Segmentation

Pre-treatment MRI and CT scans were acquired as part of the standard of care. MRI scans were obtained from either a 1.5 T Signa system (GE Healthcare, Chicago, IL, USA) or a 1.5 T Aera system (Siemens Healthcare, Erlangen, Germany), both equipped with an 8-channel breast coil. The sequences analyzed included T2 and CE-T1 images. CT scans were performed with BrightSpeed or LightSpeed scanners (GE Healthcare, Chicago, IL, USA) using a chest/abdomen/pelvis protocol with contrast enhancement. Detailed acquisition parameters for MRI and CT are provided in Supplementary Tables S1 and S2. As per study inclusion criteria, all patients had CE-T1, T2, and CT images available for analysis.

**Figure 1:**
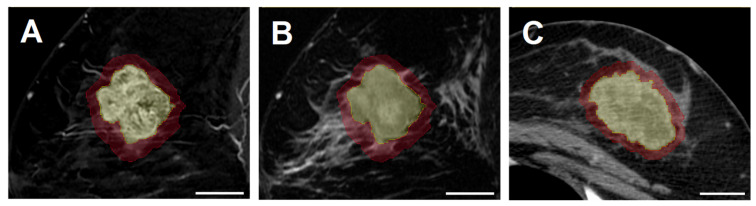
Examples of intratumoral (yellow) and peritumoral (red) segmentations on (**A**) contrast-enhanced T1-weighted MRI (CE-T1), (**B**) T2-weighted MRI, and (**C**) contrast-enhanced CT images. All images are from the same patient, with (**A**,**B**) displayed in the sagittal plane and (**C**) in the axial plane. White scale bars at the bottom right of each image correspond to 2.5 cm.

Tumor segmentation was carried out manually using 3D Slicer (v5.2.1), an open-source software for medical image analysis. The breast tumor was contoured slice by slice for volumetric intratumoral segmentation in the sagittal plane for CE-T1 images and in the axial plane for CT images. T2 images were co-registered to CE-T1 by default, and the same intratumoral contours were applied. To characterize the surrounding tumor environment, peritumoral segmentation was generated by isotropically expanding the tumor boundary by 5 mm [23,33]. Portions of the peritumoral areas extending into the chest wall or skin were excluded to confine the analysis to breast tissue. All segmentations were reviewed independently by a radiologist and a radiation oncologist for validation. Examples of intratumoral and peritumoral segmentations on CE-T1, T2, and CT are presented in Figure 1.

### 2.5. Radiomic Feature Extraction

Preprocessing steps were applied prior to radiomic feature extraction to reduce acquisition-related variability, standardize image quality, and enhance feature reproducibility and stability. Different preprocessing strategies were employed for MRI and CT because MRI signal intensities are inherently non-standard and relative, while CT voxel values are quantified in Hounsfield Units (HU), a standardized physical scale. For MRI data (T2 and CE-T1), N4 bias field correction was performed to reduce intensity inhomogeneity, followed by Z-score normalization to minimize inter-scan variability, and gray level discretization with a fixed bin count of 128. For CT images, gray level discretization with a fixed bin width of 2 was applied without normalization, in order to preserve the physical meaning of HU. In addition, all images were resampled to an isotropic voxel spacing of 1 mm × 1 mm × 1 mm.

Radiomic features were extracted using the PyRadiomics package (v3.0.1) implemented in Python (v3.8.8) [34]. From each intratumoral and peritumoral region of each image, 14 shape features, 18 first-order features, and 75 second-order features were extracted. Shape features characterized the structural and volumetric properties of the ROIs [35]. First-order features describe the statistical distribution of voxel intensities within the segmented region [35]. Second-order features, also known as texture features, captured relationships between neighboring voxels and patterns of intensity variation [35]. The texture feature set comprised 24 Gray Level Co-occurrence Matrix (GLCM) features, 16 Gray Level Run Length Matrix (GLRLM) features, 16 Gray Level Size Zone Matrix (GLSZM) features, 14 Gray Level Dependence Matrix (GLDM) features, and 5 Neighboring Gray Tone Difference Matrix (NGTDM) features. From the two MRI sequences, a total of 400 features were extracted, including 28 shape, 72 first-order, and 300 texture features across intratumoral and peritumoral regions. It should be noted that shape features are independent of voxel intensity and were therefore extracted only once per segmentation rather than separately for each MRI sequence. From CT, 214 radiomic features were extracted, comprising 28 shape, 36 first-order, and 150 texture features across intratumoral and peritumoral regions. In total, 614 handcrafted features were extracted per patient. A full list of radiomic features is provided in Supplementary Table S3.

### 2.6. Deep Feature Extraction

Unsupervised deep feature extraction was implemented using a transfer learning approach with MedicalNet, which provides pre-trained weights for 3D ResNet architectures trained on large-scale medical imaging datasets [30]. For each patient, MR and CT images were pre-processed through isotropic resampling, segmentation-based cropping, and intensity normalization. Cropped volumes were subsequently resized to 64 × 64 × 64 voxels to standardize patch size. Four pre-trained MedicalNet backbones were tested, corresponding to ResNet10, ResNet18, ResNet34, and ResNet50 architectures, where the numerical designation indicates the total number of layers in the network. The processed images were passed through the ResNet architectures initialized with their corresponding MedicalNet pre-trained weights, and the global average pooling output of the final convolutional block was retained as the deep feature vector. For each image and segmentation, ResNet10, ResNet18, and ResNet34 yielded 512 features, whereas ResNet50 yielded 2048 features. The feature numbers correspond to the number of channels in the final convolutional block of each architecture. As features were derived separately for intratumoral and peritumoral segmentations across CE-T1, T2 and CT images, six region-specific feature sets were generated per patient. Therefore, a total of 3072 features were extracted from ResNet10, ResNet18 and ResNet34, while a total of 12,288 features were extracted from ResNet50.

### 2.7. Machine Learning

Five models were developed using handcrafted features and deep features from four ResNet architectures, each combined with the seven clinical features. As ground-truth labels, non-pCR and non-response were assigned as negative classes, while pCR and response were assigned as positive classes. Model training and performance evaluation were carried out using a repeated partitioning approach consisting of ten independent stratified splits that conserves the proportion between the majority and minority classes. In each partition, 80% of the patients were randomly assigned for training and the remaining 20% were assigned for hold-out testing. All preprocessing, feature selection, and model optimization steps were performed only within the training data to prevent information leakage, whereas performance evaluation was carried out on the corresponding hold-out test set. Performance was quantified using balanced accuracy, sensitivity, specificity, precision, F1-score, and the area under the receiver operating characteristic curve (AUC). The average and standard deviation of these metrics across the ten partitions were reported to provide a robust estimate of generalization performance.

Two preprocessing steps were implemented before feature selection. Firstly, ComBat harmonization was applied independently within each imaging modality to address scanner-related heterogeneity. ComBat harmonization models scanner or protocol effects as additive bias and multiplicative scaling in feature distributions. It estimates these parameters for each scanner using an empirical Bayesian model and then rescales the features so that all scanners share a unified statistical distribution while retaining inter-patient biological variability. This framework has been validated in both phantom [36] and clinical imaging [37,38] studies, demonstrating that ComBat reduces scanner-specific noise, enhances reproducibility, and improves classification performance. Subsequently, feature values were standardized using robust scaling to reduce the influence of outliers. A three-stage feature reduction strategy was then applied to reduce dimensionality and mitigate overfitting. In the first stage, the Minimum Redundancy Maximum Relevancy (mRMR) algorithm was applied separately to MRI features (CE-T1 and T2 combined) and to CT features, with each subset limited to 10% of the cohort size. In the second stage, the selected MRI and CT features were concatenated with clinical variables, and another round of mRMR was conducted, again feature numbers were limited to 10% of the cohort size. Lastly, recursive feature elimination (RFE) with an Extreme Gradient Boosting (XGBoost) classifier (v3.0.4) was employed to further refine the subset of predictors. RFE was implemented with five-fold cross-validation within the training set and performed independently within each partition. The significance of selected features was evaluated by recording the frequency with which they appeared across the ten iterations. The final classification models were trained using XGBoost, a gradient-boosted tree algorithm widely used in medical imaging research due to its accuracy and efficiency. Hyperparameters were optimized through grid search with five-fold cross-validation in the training data. the Synthetic Minority Over-sampling Technique for Nominal and Continuous features (SMOTENC) was applied within each training fold after partitioning, ensuring that synthetic samples were generated exclusively from the training data. This method generates synthetic samples of the minority class by interpolating between existing cases, thereby balancing the class distribution while preserving data characteristics [39]. The hyperparameter settings are detailed in Supplementary Table S4.

### 2.8. Statistical Analysis

Statistical analyses were conducted to compare the distributions of clinical variables and selected radiomic features between responsive and non-responsive groups under the two response criteria. Continuous variables were analyzed using an independent-samples *t*-test or a Mann–Whitney U test, based on the normality determined by the Shapiro–Wilk test. Categorical variables were analyzed using Fisher’s exact test. Differences in model performance between the best-performing model and the other models were evaluated using paired two-tailed *t*-tests. Across all analyses, a *p*-value less than 0.05 was considered statistically significant.

## 3. Results

### 3.1. Patient Characteristics

Based on eligibility, 177 patients were included in the study. For MRI, 153 patients were imaged using a GE Signa scanner, while 24 patients were imaged with a Siemens Aera scanner. For CT imaging, 127 patients were scanned with a GE Lightspeed system and 50 patients with a GE Brightspeed system. According to Fisher’s exact test, scanner type was not significantly associated with treatment response under either criterion. The potential impact of scanner-related variability on the extracted features was mitigated through ComBat harmonization, which standardized feature distributions across scanners while preserving biological variability.

The distributions of clinical variables between responsive and non-responsive groups are summarized in Table 1 and Table 2 for criterion 1 and criterion 2, respectively. For citerion 1, 37 patients (20.9%) and 140 patients (79.1%) were categorized as pCR and non-pCR, respectively. For criterion 2, the cohort consisted of 124 responders (70.0%) and 53 non-responders (30.0%). Statistical analysis demonstrated that the receptor status (ER, PR and HER2) and histological grade differed significantly between response groups in both criteria. In addition, under criterion 1, nodal status was significant, and tumor size demonstrated a trend toward significance (*p* = 0.073). Under criterion 2, age approached significance (*p* = 0.064).

### 3.2. Classification Results

**Figure 2:**
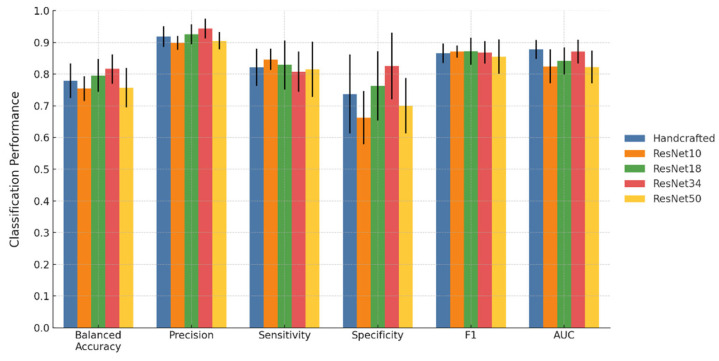
Performance comparison of handcrafted features and deep features derived from ResNet10, ResNet18, ResNet34 and ResNet50 predicting pCR vs. non-pCR (Criterion 1). Error bars represent the standard deviation obtained from ten independent partitions.

**Figure 3:**
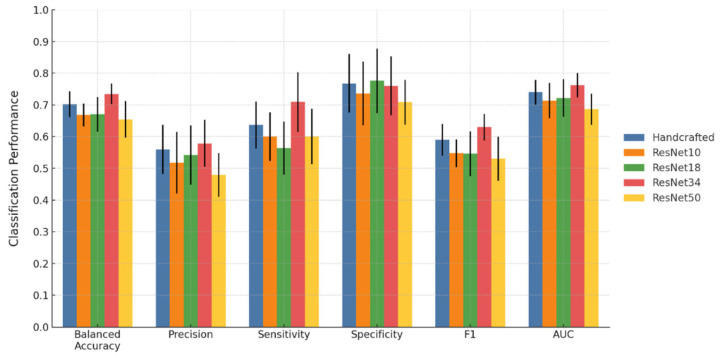
Performance comparison of handcrafted features and deep features derived from ResNet10, ResNet18, ResNet34 and ResNet50 (Criterion 2). Error bars represent the standard deviation obtained from ten independent partitions.

The classification performance of the five models is shown in Figure 2 and Figure 3 for criterion 1 and criterion 2, respectively. Detailed results with standard deviations are provided in Supplementary Tables S5 and S6. Across both criteria, deep features extracted from ResNet34 yielded the strongest overall performance. Under criterion 1, the ResNet34 model achieved a balanced accuracy of 81.6%, precision of 94.3%, sensitivity of 80.7%, specificity of 82.5%, F1-score of 0.868, and an AUC of 0.871. Its balanced accuracy, precision, and specificity were significantly higher than those of the handcrafted, ResNet10, and ResNet50 models, while its AUC was significantly higher than those of the ResNet10, ResNet18, and ResNet50 models. Under criterion 2, the ResNet34 model achieved a balanced accuracy of 73.5%, precision of 57.9%, sensitivity of 70.9%, specificity of 76%, F1-score of 0.630, and an AUC of 0.762. For this criterion, the ResNet34 model achieved significantly higher balanced accuracy and F1-score compared with all other models. Its precision was significantly higher than that of the ResNet50 model, its recall was significantly higher than those of the ResNet10, ResNet18, and ResNet50 models, and its AUC was significantly higher than those of the ResNet18 and ResNet50 models. Results of paired two-tailed tests comparing ResNet34 with the other models are provided in Supplementary Tables S7 and S8.

### 3.3. Features Selected

As 10 independent data partitions were used during training, this produced 10 distinct sets of selected features. Therefore, the significance of each feature was inferred from its frequency of selection in the ResNet34 model, which provided the best overall classification performance. Table 3 and Table 4 list the features that were selected in at least five partitions for criterion 1 and criterion 2, respectively.

Since 177 patients were included in the study, 18 features, representing approximately 10% of the cohort size, were initially selected by mRMR, and subsequent RFE could further reduce the number of selected features to limit dimensionality and mitigate the risk of overfitting. Under criterion 1, 67 unique features were selected across 10 partitions, with an average of 15.9 per partition. Of these, 37 appeared in only one partition, whereas 11 recurred in at least five partitions. The recurrent set consisted of five clinical features and six deep features, including one derived from CE-T1, three from T2, and two from CT. Among six deep features, three originated from intratumoral regions and three from peritumoral regions. Under criterion 2, 73 unique features were selected across 10 partitions, with an average of 15.6 per partition. Of these, 44 appeared in a single partition, while nine recurred in at least five partitions. This group consisted of four clinical variables and five deep features, including one from CE-T1, two from T2, and two from CT. Among the five deep features, two were derived from intratumoral regions and three from peritumoral regions.

**Figure 4:**
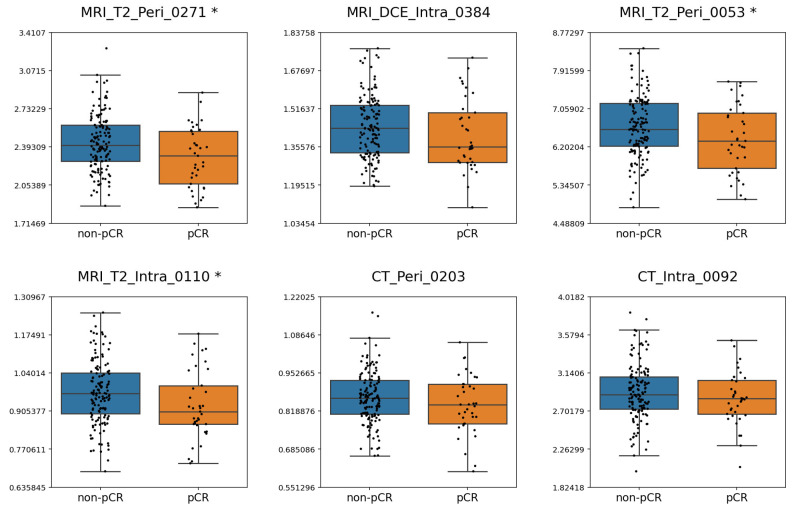
Boxplots illustrating the distribution of the most frequently selected features under criterion 1. Features showing significant differences between the pCR and non-pCR groups (*p* < 0.05) are marked with an asterisk (*) beside their names.

**Figure 5:**
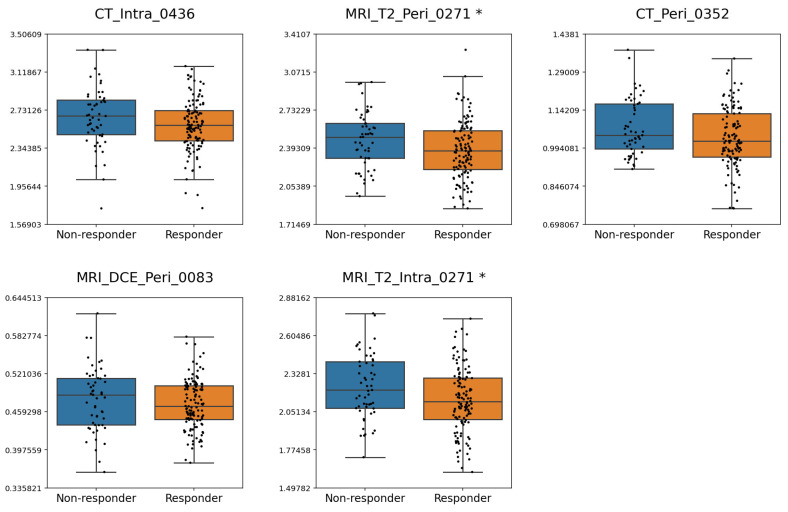
Boxplots illustrating the distribution of the most frequently selected features under criterion 2. Features showing significant differences between the responders and non-responders (*p* < 0.05) are marked with an asterisk (*) beside their names.

Boxplots illustrating the distribution of frequently selected deep features across response groups for criterion 1 and criterion 2 are shown in Figure 4 and Figure 5, respectively. Depending on the normality of each feature, either an independent samples *t*-test or a Mann–Whitney U test was applied to compare groups. For criterion 1, MRI_T2_Peri_0271, MRI_T2_Peri_0053, and MRI_T2_Intra_0110 exhibited significant differences between the pCR and non-pCR groups. For criterion 2, MRI_T2_Peri_0271 and MRI_T2_Intra_0271 differed significantly between responders and non-responders. Across both criteria, lower values of MRI_T2_Peri_0271 were consistently associated with response.

## 4. Discussion

In this study, a deep feature extraction framework was applied to pre-treatment MRI and CT scans, together with clinical information, to model response to neoadjuvant chemotherapy in breast cancer. Rather than relying solely on handcrafted radiomic features, this approach used convolutional neural networks pre-trained on large-scale medical imaging datasets to capture complex representations from both intratumoral and peritumoral regions. Two clinically relevant definitions of response were examined: one distinguishing pCR from non-pCR, and the other differentiating complete or partial response from stable or progressive disease. By incorporating multimodal and multiregional imaging features with established clinical predictors, the analysis demonstrates the potential of deep learning-based features to complement conventional radiomics and provide additional value for treatment response assessment.

Statistical analysis of the clinical variables indicated that histologic grade and receptor status were significantly associated with response in both criteria; tumors with high histologic grade, ER/PR negativity, and HER2 positivity were more likely to respond to NAC. This finding is consistent with the clinical understanding that more aggressive disease, characterized by these variables, is more responsive to anthracycline- and taxane-based regimens that preferentially target highly proliferative cells. Furthermore, the availability of subtype-specific treatments, such as targeted therapy for HER2-enriched breast cancer and immunotherapy for triple-negative breast cancer, enhances tumor response. In addition to these factors, nodal status was significantly associated with pCR, consistent with its definition requiring absence of nodal disease. Although neither age nor initial tumor size reached statistical significance, tumor size (*p* = 0.073) and age (*p* = 0.064) showed trend towards statistical significance for criterion 1 and criterion 2, respectively.

In this study, handcrafted radiomic features and deep features extracted from ResNet10, ResNet18, ResNet34, and ResNet50 were evaluated. In addition, clinical features were included in all feature sets for analysis, as previous MRI [40] and CT [41] studies demonstrated the utility of combining clinical and radiomic features to improve classification performance. Among the five models, the ResNet34 model achieved the best overall classification performance in both criteria. Most notably, the ResNet34 model outperformed the handcrafted model with significantly higher balanced accuracy, precision, and specificity under criterion 1. For criterion 2, the ResNet34 model achieved significantly higher balanced accuracy and F1-score. No significant differences were observed in the remaining performance metrics. This performance advantage demonstrated the utility of deep features for predicting treatment response. Deep features are quantitative image representations from convolutional neural networks pre-trained on large imaging datasets, with shallow layers capturing basic textures and deeper layers encoding higher-order structural patterns. Therefore, deep features may capture multi-scale patterns beyond the predefined mathematical definitions of handcrafted features, providing more comprehensive imaging representations that translate into superior classification performance. It is also important to note that other deep feature models involving ResNet10, ResNet18, and ResNet50 did not demonstrate superiority to the handcrafted feature model. ResNet10 and ResNet18 are shallow networks with 10 and 18 layers, each producing 512-dimensional feature vectors. ResNet34 increases depth to 34 layers while retaining 512-dimensional feature vectors, enabling richer hierarchical representations. Even though the number of layers differs among the three architectures, the number of final features remains the same because they share an identical overall design, with the last stage fixed at 512 channels prior to global average pooling. ResNet50 extends to 50 layers and generates 2048-dimensional feature vectors, greatly expanding the feature space. It is postulated that the weaker performance of ResNet10 and ResNet18 likely reflects their limited depth, which restricts extraction of complex multi-scale patterns and yields less discriminative features. In contrast, the high-dimensional representations from ResNet50 may introduce redundancy and overfitting, even after rigorous feature selection, particularly given the segmentation- and image-dependent extraction that expands the total feature dimension to 12,288. ResNet34 provides a balance, offering sufficient depth for higher-order abstractions while maintaining a manageable feature space, leading to more stable and discriminative representations.

The frequency of feature selection across ten partitions of training and test sets was used as an indicator of feature importance, and features selected at least five times were considered more significant. The clinical relevance of receptor status and histologic grade was highlighted again, as these variables were consistently selected in both criteria, with the exception of PR under criterion 2. In criterion 1, PR and HER2 status were both selected ten times, while ER status and histologic grade were selected seven times. In criterion 2, HER2 status and histologic grade were selected nine times while ER status was selected seven times. Notably, age, which was not statistically significant under either criterion, was selected five times and ten times for criterion 1 and criterion 2, respectively. This reflects that predictive relevance in multivariate machine learning models does not always correspond directly to univariate statistical associations [42].

In addition to these clinical predictors, deep features from CE-T1, T2, and CT were frequently selected, underscoring their complementary value for treatment response prediction. For criterion 1, six deep features were selected, including one from CE-T1, three from T2, and two from CT, with equal intratumoral and peritumoral representation. For criterion 2, five deep features were selected, including one from CE-T1, two from T2, and two from CT, with two intratumoral and three peritumoral features. The distribution of frequently selected deep features across CE-T1, T2, and CT suggests that each modality captures distinct biological information. CE-T1 captures contrast uptake, reflecting tumor vascularity, perfusion, and permeability, which are related to angiogenesis and necrosis [43]. T2 represents tissue water content, making it sensitive to edema, cystic or necrotic change, and stromal composition [44]. CT characterizes tissue density and enhancement, thereby reflecting vascularity as well as heterogeneity arising from necrosis, fibrosis, or calcification [45]. Furthermore, intratumoral features capture the internal heterogeneity of the tumor, such as cellularity, necrosis, and vascular patterns, whereas peritumoral features reflect the surrounding microenvironment, including stromal reaction, edema, and vascular remodeling. Together, these intratumoral and peritumoral deep features from multiple modalities integrate vascular, compositional, and density-based information, yielding a more comprehensive characterization of the tumor and its microenvironment relevant to treatment response prediction.

Several studies have investigated the utilization of MRI- or CT-based deep learning and deep feature approaches for response prediction in breast cancer. Li et al. conducted a multicenter investigation across four institutions involving 1048 patients [46]. A predictive model was developed that combined handcrafted radiomic features with ResNet50-derived deep features from pre- and mid-treatment MRI to classify residual cancer burden (RCB) using an SVM classifier. When evaluated on pre-treatment MRI, the model yielded AUCs of 0.817, 0.787, and 0.809 across three independent external validation cohorts for distinguishing RCB 0–I from RCB II–III. For separating RCB 0–II from RCB III, the model achieved AUCs of 0.833, 0.819, and 0.801. Peng et al. utilized pretreatment CE-T1 to compare a ResNeXt50-based deep learning model with handcrafted radiomics for pCR in 356 breast cancer patients [47]. Upon five-fold cross-validation, the integrative deep learning model that combined imaging, kinetic parameters, and molecular information achieved an AUC of 0.83, outperforming the integrative handcrafted radiomic model using a linear discriminant analysis (LDA) classifier, which achieved an AUC of 0.781. Dammu et al. developed ResNet-based deep learning models incorporating longitudinal multiparametric MRI and clinical information to predict pCR in 155 breast cancer patients [48]. With pre-treatment MRI combined with clinical information, an accuracy of 0.81 and an AUC of 0.83 were achieved. Li et al. investigated the extraction of deep features using UCTransNet and handcrafted radiomic features extracted with Pyradiomics from pre- and early-treatment CE-T1, along with clinical information, to predict pCR after NAC [49]. Using a 70% training and 30% hold-out test split, the combined model from the pre-treatment imaging data achieved an AUC of 0.738. Moslemi et al. evaluated 117 patients with LABC using pretreatment CT with multiple deep learning architectures, including transfer learning CNNs (VGG16/19, ResNet50/101/152, InceptionV3, Xception) and a Vision Transformer trained end-to-end, to predict NAC response [50]. With 10 iterations of a 70:30 split for training and testing, the Transformer ViT model achieved a balanced accuracy of 66%, outperforming the other CNN-based models. However, that study utilized CNNs pre-trained on the ImageNet-1k dataset, which consists of generic natural images rather than medical imaging. As a result, the transfer learning applied in this context may not adequately capture the complexity of 3D medical imaging. Falou et al. investigated 174 patients with LABC using pre-treatment quantitative ultrasound (QUS) parametric maps for predicting NAC response [51]. A transfer learning approach with ResNet50V2 was used for deep feature extraction from 2D intratumoral and peritumoral segmentations. Using a support vector machine (SVM) classifier, the spectral slope-based model achieved the best performance on an independent test set with a balanced accuracy of 86% for identifying non-responders.

Collectively, previous studies demonstrate the potential of deep learning and deep feature approaches for predicting NAC response, although most have been limited to single-modality analyses, intratumoral segmentation, or models pre-trained on natural image datasets. The present study introduces a multi-modal and multi-segmental framework that integrates intratumoral and peritumoral deep features from CE-T1- and T2-weighted MRI and contrast-enhanced CT, allowing a more comprehensive characterization of tumor heterogeneity and the surrounding tissue environment. MedicalNet-based three-dimensional architectures pre-trained on large medical imaging datasets were used to extract volumetric features that better reflect the spatial complexity of clinical imaging. In addition, two independent response criteria, pathological complete response and a RECIST-based classification, were examined to evaluate the consistency of predictive performance across distinct clinical endpoints.

The study here has several limitations that should be acknowledged. First, the dataset was derived from a single institution and included a relatively small number of patients, which limits the generalizability of the findings. Although repeated cross-validation with ten independent partitions aimed to provide robust and unbiased performance estimates, it cannot fully account for systematic biases inherent to a single-center dataset. To address this, future studies should prioritize external validation on larger, multi-institutional cohorts collected with heterogeneous imaging protocols, which would allow a more rigorous assessment of model robustness in diverse clinical environments. Expanding the cohort size would also create opportunities for multi-class classification of non-responders, partial responders, and complete responders, while ensuring adequate sample sizes for each subgroup. Furthermore, a larger and multi-institutional cohort would enable stratified modeling by molecular subtype, as therapeutic response and prognosis differ substantially across Luminal A, Luminal B, HER2-enriched, and triple-negative cancers. In addition, such a cohort would allow examination of potential differences in treatment response between patients receiving the standard and dose-dense regimens, as well as potentially other non-standard chemotherapy protocols.

## 5. Conclusions

In conclusion, this study evaluated a deep learning-based framework for predicting response to neoadjuvant chemotherapy in breast cancer using pre-treatment MRI and CT scans together with clinical information. Among the tested architectures, ResNet34 achieved the best performance for both classifications, distinguishing pCR from non-pCR and responders from non-responders. It outperformed handcrafted radiomics as well as the other ResNet models. Through transfer learning, deep feature extraction provided intratumoral and peritumoral representations that captured greater complexity than handcrafted radiomics. These findings suggest that routinely available imaging can be utilized for multimodal deep feature extraction to complement conventional approaches and support more personalized treatment strategies by identifying patients less likely to benefit from standard NAC regimens.

## Figures and Tables

**Table 1:** Comparison of clinical characteristics between pCR and non-pCR groups (Criterion 1).

Characteristics	pCR (n = 37)	Non-pCR (n = 140)	All (n = 177)	*p* Value
Age (year)	50.2 ± 7.2	48.6 ± 10.8	48.9 ± 10.1	0.269
Initial Tumor Size (mm)	35.4 ± 16.1	42.0 ± 22.6	40.7 ± 21.6	0.073
Histologic Grade				0.003
I (%)	1 (2.7%)	9 (6.4%)	10 (5.6%)	
II (%)	8 (21.6%)	69 (49.3%)	77 (43.5%)	
III (%)	28 (75.7%)	62 (44.3%)	90 (50.8%)	
ER				<0.001
Negative (%)	24 (64.9%)	39 (27.9%)	63 (35.6%)	
Positive (%)	13 (35.1%)	101 (72.1%)	114 (64.4%)	
PR				<0.001
Negative (%)	30 (81.1%)	48 (34.3%)	78 (44.1%)	
Positive (%)	7 (18.9%)	92 (65.7%)	99 (55.9%)	
HER2				<0.001
Negative (%)	12 (32.4%)	103 (73.6%)	115 (65.0%)	
Positive (%)	25 (67.6%)	37 (26.4%)	62 (35.0%)	
Nodal Status				0.023
N0 (%)	11 (29.7%)	33 (23.6%)	44 (24.9%)	
N1 (%)	25 (67.6%)	79 (56.4%)	104 (58.8%)	
N2 (%)	0 (0%)	23 (16.4%)	23 (13.0%)	
N3 (%)	1 (2.7%)	5 (3.6%)	6 (3.4%)	

**Table 2:** Comparison of clinical characteristics between response and non-response groups (Criterion 2).

Characteristics	Response (*n* = 124)	Non-Response (n = 53)	All (n = 177)	*p* Value
Age (year)	47.9 ± 9.2	51.3 ± 11.6	48.9 ± 10.1	0.064
Initial Tumor Size (mm)	41.5 ± 22.9	38.8 ± 18.0	40.7 ± 21.6	0.798
Histologic Grade				<0.001
I (%)	7 (5.6%)	3 (5.7%)	10 (5.6%)	
II (%)	43 (34.7%)	34 64.2%)	77 (43.5%)	
III (%)	74 (59.7%)	16 (30.2%)	90 (50.8%)	
ER				<0.001
Negative (%)	54 (43.5%)	9 (17.0%)	63 (35.6%)	
Positive (%)	70 (56.5%)	44 (83.0%)	114 (64.4%)	
PR				0.008
Negative (%)	63 (50.8%)	15 (28.3%)	78 (44.1%)	
Positive (%)	61 (49.2%)	38 (71.7%)	99 (55.9%)	
HER2				<0.001
Negative (%)	70 (56.5%)	45 (84.9%)	116 (65.2%)	
Positive (%)	54 (43.5%)	8 (15.1%)	62 (34.8%)	
Nodal Status				0.647
N0 (%)	33 (26.6%)	11 (20.8%)	44 (24.9%)	
N1 (%)	73 (58.9%)	31 (58.5%)	104 (58.8%)	
N2 (%)	14 (11.3%)	9 (17.0%)	23 (13.0%)	
N3 (%)	4 (3.2%)	2 (3.8%)	6 (3.4%)	

**Table 3:** Summary of frequently selected features for Criterion 1. Features are categorized into clinical (white), CE-T1 (green), T2 (blue), and CT (yellow) features.

Features	Frequency
PR	10
HER2	10
T2_Peri_0271	10
CE-T1_Intra_0384	8
ER	7
Histologic Grade	7
T2_Peri_0053	6
T2_Intra_0110	6
CT_Peri_0203	6
Age	5
CT_Intra_0092	5

**Table 4:** Summary of frequently selected features for Criterion 2. Features are categorized into clinical (white), CE-T1 (green), T2 (blue), and CT (yellow) features.

Features	Frequency
Age	10
HER2	9
Histologic Grade	9
ER	7
CT_Intra_0436	5
T2_Peri_0271	5
CT_Peri_0352	5
CE-T1_Peri_0083	5
T2_Intra_0271	5

## Data Availability

Data are available upon request (contact the Czarnota Lab at Sunnybrook Health Sciences Centre).

## Supplementary Materials

The following supporting information can be downloaded at: https://www.mdpi.com/article/10.3390/cancers17203394/s1. Table S1. MRI acquisition parameters. Values marked with * indicate an approximate average. Table S2. CT acquisition parameters. Table S3. List of features extracted using PyRadiomics. Table S4. Hyperparameter tuning settings for XGBoost machine learning. Table S5. Performance metrics of handcrafted, ResNet10, ResNet18, ResNet34 and ResNet50 models for predicting pCR vs. non-pCR (Criterion 1). The average and standard deviation values were obtained across ten partitions. Table S6. Performance metrics of handcrafted, ResNet10, ResNet18, ResNet34 and ResNet50 models for predicting response vs. non-response (Criterion 2). The average and standard deviation values were obtained across ten partitions. Table S7. *p*-values of two-tailed *t*-test comparing classification performance between ResNet34 and other models for criterion 1. Statistical significance with *p*< 0.05 is marked with * and *p* < 0.001 is marked with **. Table S8. *p*-values of two-tailed *t*-test comparing classification performance between ResNet34 and other models for criterion 2. Statistical significance with *p* < 0.05 is marked with * and *p* < 0.001 is marked with **.
