# Supplementary material for: A Priori Prediction of Neoadjuvant Chemotherapy Response in Breast Cancer Using Deep Features from Pre-Treatment MRI and CT
Source: Cancers (Basel). 2025 Oct 21;17(20):3394. doi: 10.3390/cancers17203394 (PMC12563722; doi:10.3390/cancers17203394)
Supplement: Supplementary file 1 [file cancers-17-03394-s001.zip › cancers-3911232-supplementary.pdf]

Table S1. MRI acquisition parameters. Values marked with * indicate an approximate average.

Scanner	Patients No.	Sequence	Repetition Time (ms)	Echo Time (ms)	Slice Thickness (mm)	Flip Angle	Contrast Agent
GE 1.5T (Signa)	153	T2	2500*	76.14*	3	90°	N/A
		CE-T1	7.3*	3.5*	3	15°	Gadavist
Siemens 1.5T (Aera)	24	T2	6300*	79	3	142°*	N/A
		CE-T1	5.39	2.39	3	15°	Gadavist

Table S2. CT acquisition parameters.

Scanner	Patients No.	Tube Voltage (kVp)	Slice Thickness (mm)	Matrix Size	In-plane Pixel Size (mm)	Contrast Agent
BrightSpeed	50	120	2.0 or 2.5	512 × 512	0.5 to 1.0	Omnipaque 300
LightSpeed	127	120	2.0 or 2.5	512 × 512	0.5 to 1.0	Omnipaque 300

Table S3. List of features extracted using PyRadiomics.

Feature Class	Feature Number	Feature Name
**3D Shape**	14	Elongation
		Flatness
		Least Axis Length
		Major Axis Length
		Maximum 2D Diameter Column
		Maximum 2D Diameter Row
		Maximum 2D Diameter Slice
		Maximum 3D Diameter
		Mesh Volume
		Minor Axis Length
		Sphericity
		Surface Area
		Surface Volume Ratio
		Voxel Volume
**First Order**	18	10 Percentile
		90 Percentile
		Energy
		Entropy
		Interquartile Range
		Kurtosis
		Maximum
		Mean Absolute Deviation
		Mean
		Median
		Minimum
		Range
		Robust Mean Absolute Deviation
		Root Mean Squared
		Skewness
		Total Energy
		Uniformity
		Variance
**Gray Level Co-occurrence Matrix (GLCM)**	24	Autocorrelation
		Joint Average
		Cluster Prominence
		Cluster Shade
		Cluster Tendency
		Contrast
		Correlation

		Difference Average<br>Difference Entropy<br>Difference Variance<br>Joint Energy<br>Joint Entropy<br>Informational Measure of Correlation (IMC) 1<br>Informational Measure of Correlation (IMC) 2<br>Inverse Difference Moment (IDM)<br>Inverse Difference Moment Normalized (IDMN)<br>Inverse Difference (ID)<br>Inverse Difference Normalized (IDN)<br>Maximal Correlation Coefficient (MCC)<br>Inverse Variance<br>Maximum Probability<br>Sum Average<br>Sum Entropy<br>Sum Squares
**Gray Level Run Length Matrix (GLRLM)**	16	Gray Level Non Uniformity<br>Gray Level Non Uniformity Normalized<br>Gray Level Variance<br>High Gray Level Run Emphasis<br>Long Run Emphasis<br>Long Run High Gray Level Emphasis<br>Long Run Low Gray Level Emphasis<br>Low Gray Level Run Emphasis<br>Run Entropy<br>Run Length Non Uniformity<br>Run Length Non Uniformity Normalized<br>Run Percentage<br>Run Variance<br>Short Run Emphasis<br>Short Run High Gray Level Emphasis<br>Short Run Low Gray Level Emphasis
**Gray Level Size Zone Matrix (GLSZM)**	16	Gray Level Non Uniformity<br>Gray Level Non Uniformity Normalized<br>Gray Level Variance<br>High Gray Level Zone Emphasis<br>Large Area Emphasis<br>Large Area High Gray Level Emphasis<br>Large Area Low Gray Level Emphasis<br>Low Gray Level Zone Emphasis<br>Size Zone Non Uniformity<br>Size Zone Non Uniformity Normalized

		Small Area Emphasis<br>Small Area High Gray Level Emphasis<br>Small Area Low Gray Level Emphasis<br>Zone Entropy<br>Zone Percentage<br>Zone Variance
**Gray Level Dependence Matrix (GLDM)**	14	Dependence Entropy<br>Dependence Non Uniformity<br>Dependence Non Uniformity Normalized<br>Dependence Variance<br>Gray Level Non Uniformity<br>Gray Level Variance<br>High Gray Level Emphasis<br>Large Dependence Emphasis<br>Large Dependence High Gray Level Emphasis<br>Large Dependence Low Gray Level Emphasis<br>Low Gray Level Emphasis<br>Small Dependence Emphasis<br>Small Dependence High Gray Level Emphasis<br>Small Dependence Low Gray Level Emphasis
**Neighbouring Gray Tone Difference Matrix (NGTDM)**	5	Busyness<br>Coarseness<br>Complexity<br>Contrast<br>Strength

Table S4. Hyperparameter tuning settings for XGBoost machine learning.

Hyperparameter	Value
learning_rate	0.05 and 0.1
eval_metric	auc
max_depth	3 and 4
min_child_weight	1 and 2
subsample	0.8 and 1.0
colsample_bytree	0.8 and 1.0
lambda	1 and 5
alpha	0 and 1
gamma	0 and 0.1
n_estimators	500

Table S5. Performance metrics of handcrafted, ResNet10, ResNet18, ResNet34 and ResNet50 models for predicting pCR vs. non-pCR (Criterion 1). The average and standard deviation values were obtained across ten partitions.

Feature Set	Balanced Accuracy (%) ± SD	Precision (%) ± SD	Sensitivity (%) ± SD	Specificity (%) ± SD	F1 ± SD	AUC ± SD
Handcrafted	77.9 ± 5.5	91.8 ± 3.3	82.1 ± 5.8	73.8 ± 12.4	0.866 ± 0.031	0.878 ± 0.030
ResNet10	75.4 ± 3.9	89.8 ± 2.2	84.6 ± 3.4	66.3 ± 8.4	0.871 ± 0.019	0.825 ± 0.053
ResNet18	79.6 ± 5.2	92.6 ± 3.1	82.9 ± 7.7	76.3 ± 10.9	0.872 ± 0.043	0.841 ± 0.043
ResNet34	81.6 ± 4.6	94.3 ± 3.1	80.7 ± 6.3	82.5 ± 10.5	0.868 ± 0.035	0.871 ± 0.037
ResNet50	75.7 ± 6.2	90.5 ± 2.7	81.4 ± 8.7	70.0 ± 8.7	0.855 ± 0.054	0.822 ± 0.052

Table S6. Performance metrics of handcrafted, ResNet10, ResNet18, ResNet34 and ResNet50 models for predicting response vs. non-response (Criterion 2). The average and standard deviation values were obtained across ten partitions.

Feature Set	Balanced Accuracy (%) ± SD	Precision (%) ± SD	Sensitivity (%) ± SD	Specificity (%) ± SD	F1 ± SD	AUC ± SD
Handcrafted	70.2 ± 4.1	56 ± 7.8	63.6 ± 7.4	76.8 ± 9.2	0.590 ± 0.05	0.74 ± 0.039
ResNet10	66.8 ± 3.6	51.8 ± 9.7	60 ± 7.7	73.6 ± 10	0.547 ± 0.043	0.713 ± 0.056
ResNet18	67 ± 5.4	54.2 ± 9.3	56.4 ± 8.4	77.6 ± 10.2	0.546 ± 0.071	0.721 ± 0.059
ResNet34	73.5 ± 3.3	57.9 ± 7.4	70.9 ± 9.4	76.0 ± 9.2	0.630 ± 0.041	0.762 ± 0.038
ResNet50	65.4 ± 5.8	47.9 ± 6.9	60 ± 8.8	70.8 ± 7.1	0.531 ± 0.069	0.686 ± 0.049

Table S7. *p*-values of two-tailed t-test comparing classification performance between ResNet34 and other models for criterion 1. Statistical significance with $p < 0.05$ is marked with * and $p < 0.001$ is marked with **

Comparison	Balanced Accuracy	Precision	Sensitivity	Specificity	F1	AUC
Handcrafted	0.036 *	0.046 *	0.423	0.045 *	0.752	0.647
ResNet10	0.003 *	0.002 *	0.162	0.004 *	0.822	0.029 *
ResNet18	0.291	0.244	0.217	0.213	0.532	0.007 *
ResNet50	0.046 *	0.030 *	0.858	0.042 *	0.561	0.013 *

Table S8. *p*-values of two-tailed t-test comparing classification performance between ResNet34 and other models for criterion 2. Statistical significance with $p < 0.05$ is marked with * and $p < 0.001$ is marked with **

Comparison	Balanced Accuracy	Precision	Sensitivity	Specificity	F1	AUC
Handcrafted	0.037 *	0.634	0.137	0.879	0.040 *	0.171
ResNet10	0.0002 **	0.070	0.013 *	0.509	0.0002 **	0.029 *
ResNet18	0.004 *	0.321	0.001 *	0.670	0.004 *	0.105
ResNet50	0.001 *	0.009 *	0.024 *	0.221	0.0007 **	0.006 *
